# Atypical Volar Wrist Ganglion Originating From the Dorsal Joint Capsule: A Case Report With Diagnostic and Management Insights

**DOI:** 10.7759/cureus.86132

**Published:** 2025-06-16

**Authors:** Hussain A Alradwan, Muath S Alghamdi

**Affiliations:** 1 Orthopedics Surgery, Almoosa Specialist Hospital, Al-Ahsa, SAU

**Keywords:** case report, diagnosis, dorsal joint capsule, ganglion cysts, management, volar wrist ganglion

## Abstract

Wrist ganglion cysts are common benign lesions typically originating dorsally or volarly. While they typically present dorsally, volar ganglia originating from the dorsal aspect are exceptionally rare. Hence, this paper reports the case of a 26-year-old female with a volar wrist ganglion. Intraoperatively, it was found to originate dorsally. This case highlights the importance of meticulous surgical exploration to identify the true origin of ganglion cysts.

## Introduction

Ganglion cysts are fluid-filled masses that arise near joint capsules or tendon sheaths, often presenting as asymptomatic swellings but occasionally causing pain or functional impairment. Ganglion cysts most commonly develop in the wrist. Their exact etiology remains uncertain, though repetitive microtrauma and mucoid degeneration have been implicated [1]. The complexity of these lesions often necessitates advanced imaging for precise localization, particularly when deeper extensions or unusual anatomical courses are suspected [2-4].

While ganglion cysts frequently present on the dorsal wrist, volar ganglia account for a smaller subset and pose additional considerations due to their proximity to neurovascular structures, particularly the radial artery [5-7]. Exerting pressure on the nearby neurovascular structures could lead to atypical symptoms such as radiating pain, paresthesia, or functional impairment [8,9]. Volar ganglia usually develop at the volar radiocarpal joint, specifically within the interval separating the radioscaphocapitate and long radiolunate ligaments. However, cases originating from the dorsal structures are rare, presenting unique diagnostic and surgical challenges due to their atypical anatomical course [10,11]. In this paper, we report an atypical presentation of a volar wrist ganglion cyst originating from the dorsal wrist joint capsule, focusing on the intricacies of its diagnostic evaluation and therapeutic management.

## Case presentation

**Figure 1:**
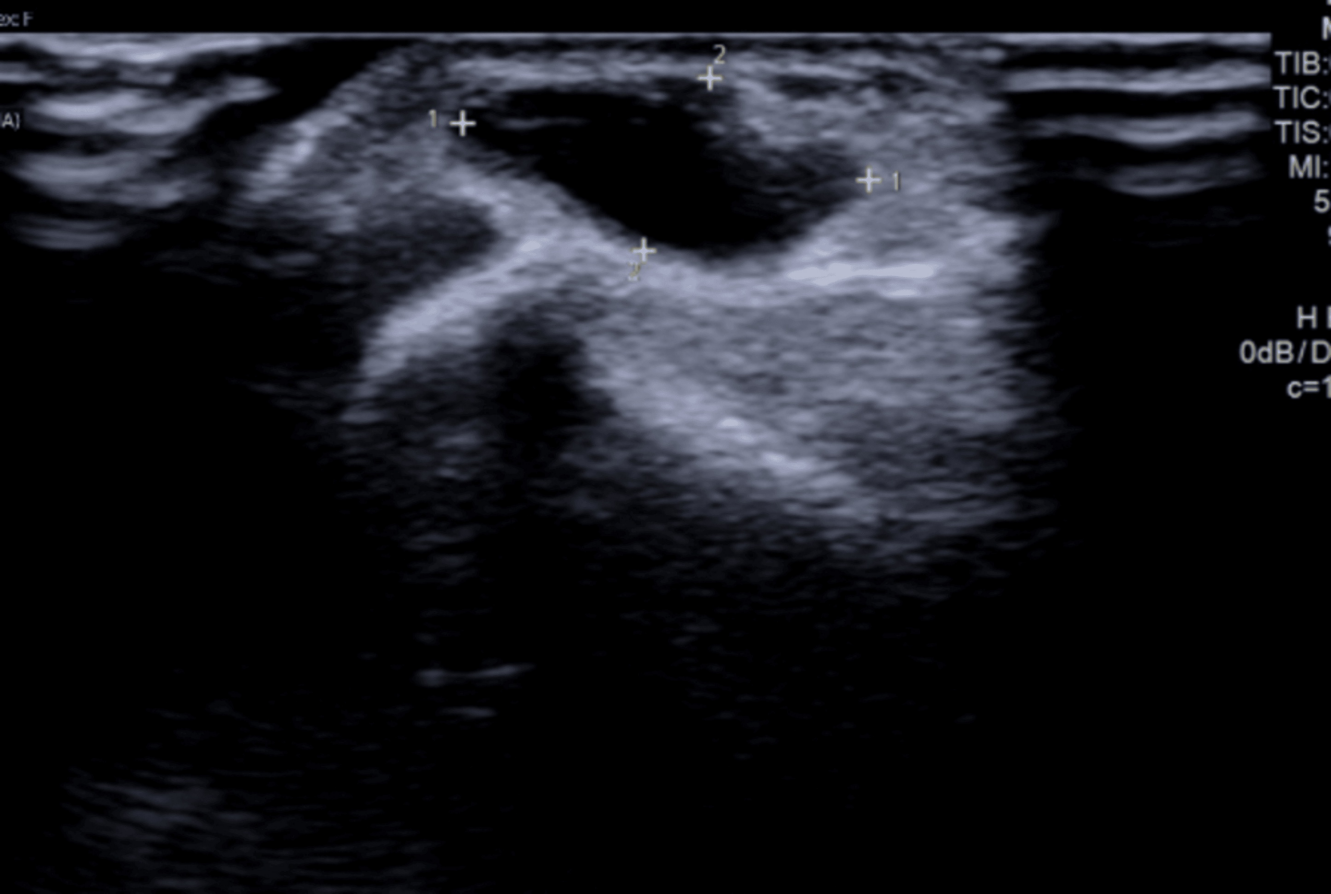
Sonographic findings of the volar ganglion of the wrist.

**Figure 2:**
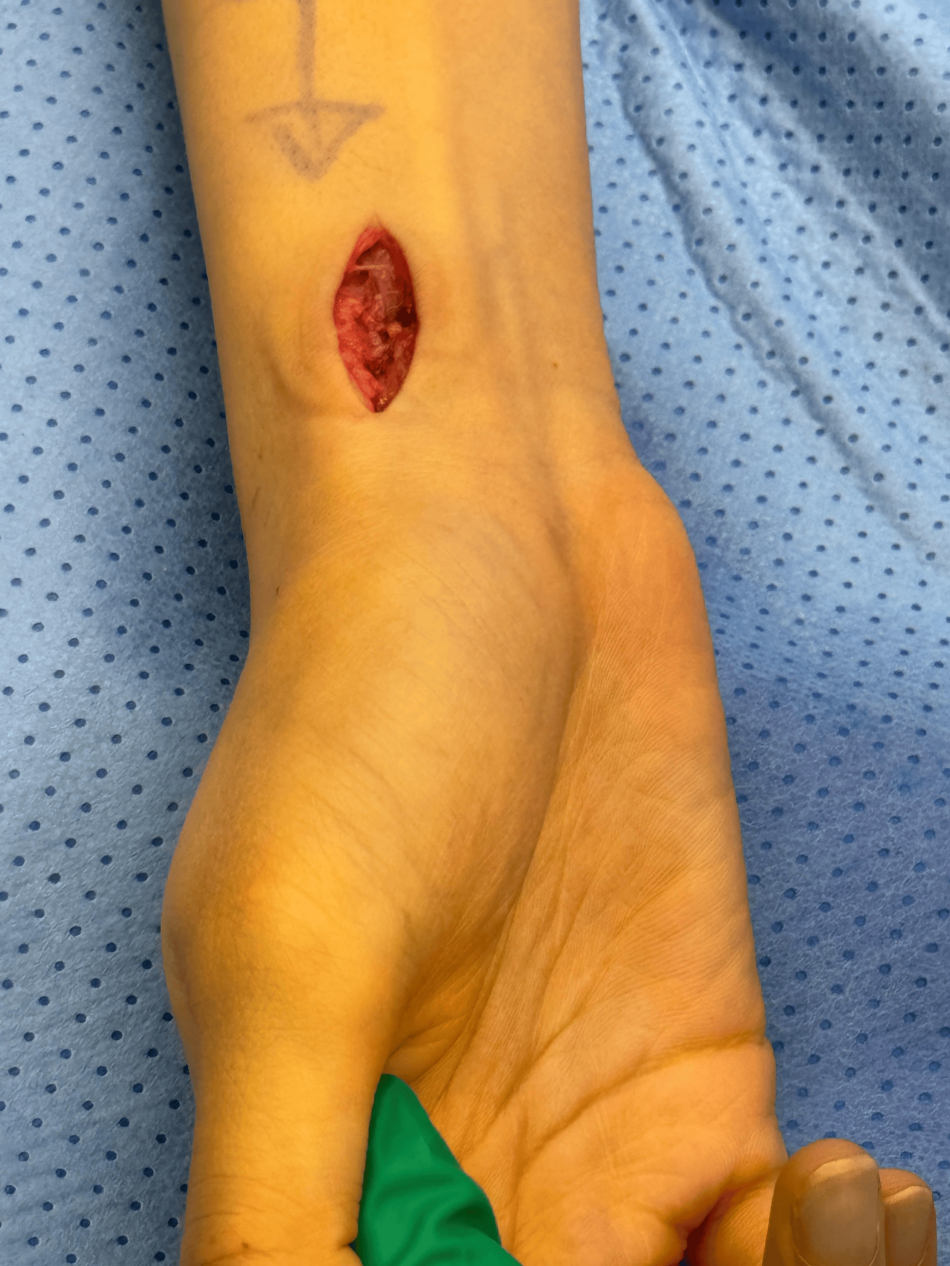
A 1-2 cm incision over the volar aspect of the wrist.

**Figure 3:**
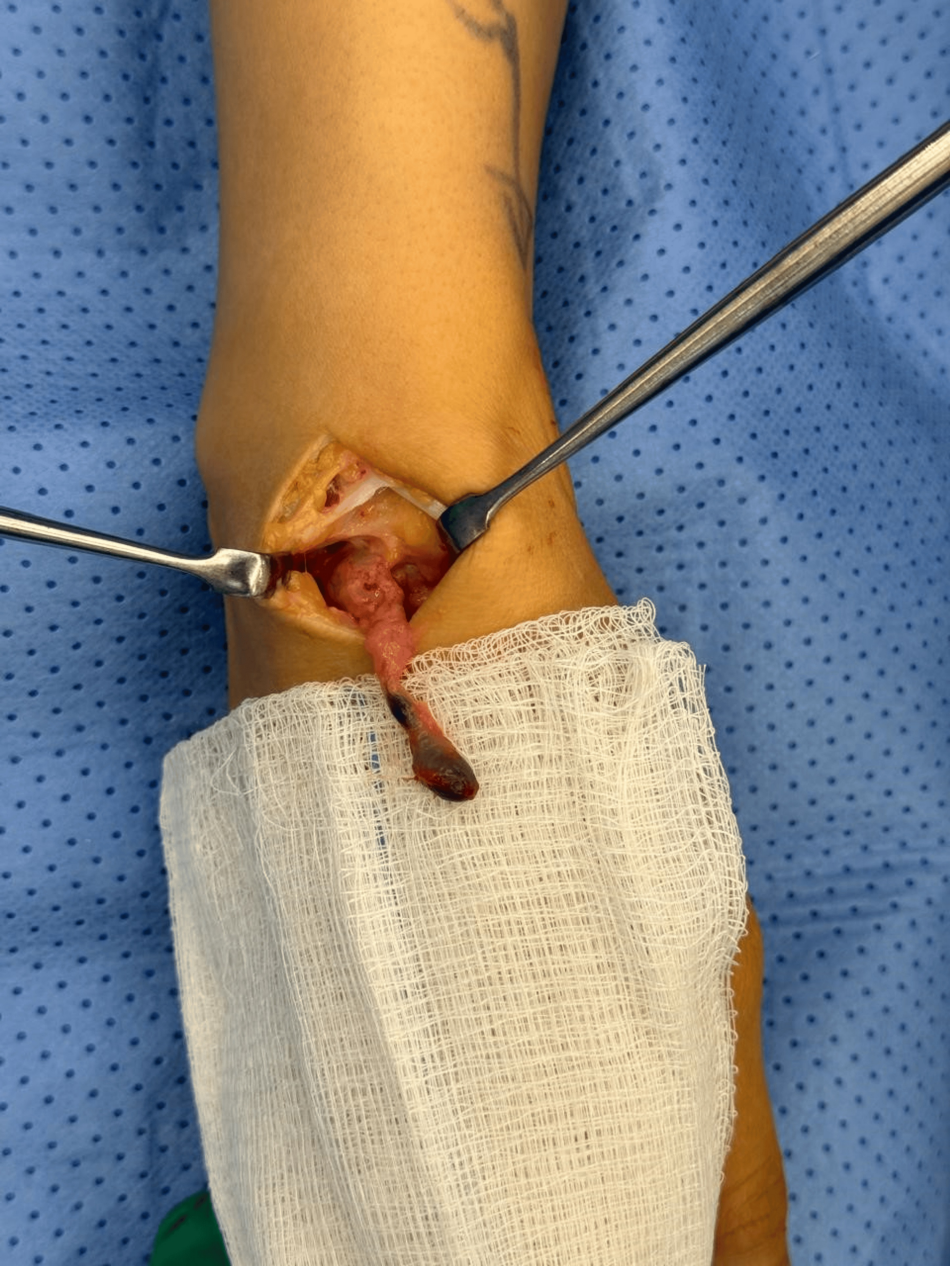
A second dorsal incision was made in line with the Lister tubercle.

**Figure 4:**
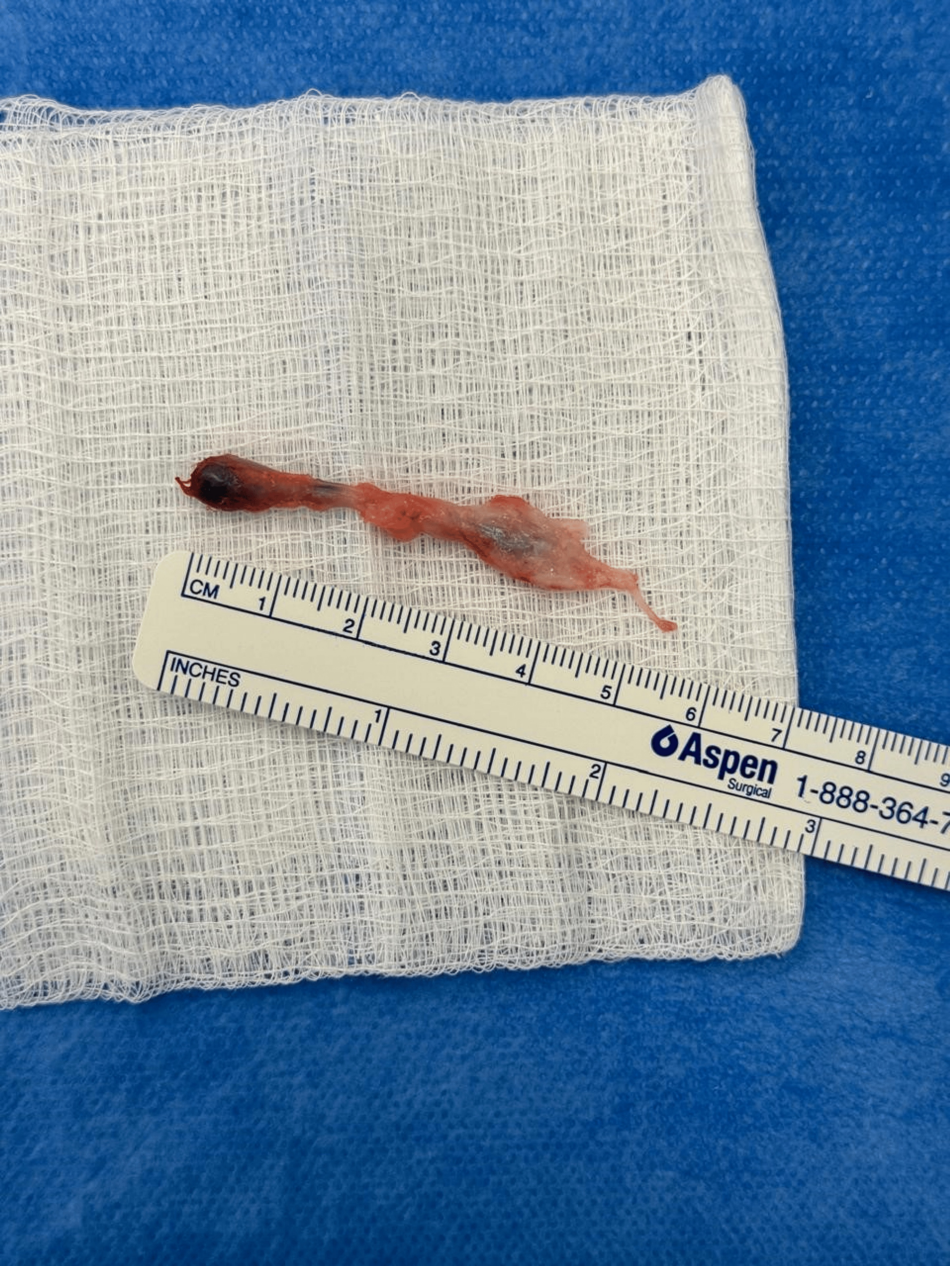
An elongated, balloon-shaped sac measuring approximately 5 cm in length was excised.

A 26-year-old female presented with a volar right wrist mass, occasionally painful for six months. The patient had no significant past medical or surgical history. The mass was non-tender, non-pulsatile, 6 mm along the radial volar wrist proximal to the flexion crease. She had a full wrist range of motion, with normal power and neurovascular exam. The diagnostic ultrasound imaging revealed a ganglion measuring 9.5 x 5.5 mm adjacent to the flexor carpi radialis tendon (Figure 1). A discussion was had with the patient regarding the available management options, including conservative observation and surgical excision. After considering each approach's potential risks and benefits, the patient opted for surgical excision as the preferred course of treatment. During surgery, the patient was positioned supine with her hand placed on an arm board, ensuring optimal surgical access and stability. For sedation and induction, 2 mg of midazolam and 10 mg of ketamine were administered. The patient also received a nerve block administered with 20 mL of 0.5% bupivacaine and 20 mL of 2% lidocaine to ensure adequate analgesia. A 2 cm longitudinal volar incision was made to dissect the ganglion (Figure 2). The radial artery was protected. The incision extended distally to trace the sac (Figure 2). Given its deep extension into the first extensor compartment and its dorsal trajectory, a second dorsal incision was required to facilitate complete exposure and excision of the cyst (Figure 3). Origin was confirmed from the dorsal wrist joint capsule, and the elongated full sac measured 5 cm (Figure 4). Standard wound closure was performed using routine surgical techniques. The final pathology reported was a ganglion cyst.

## Discussion

Volar wrist ganglia account for approximately 13% to 20% of all ganglion cysts. Although less common than dorsal wrist ganglia, they present distinct clinical challenges due to their close proximity to vital neurovascular structures, particularly the radial artery and median nerve, increasing the complexity of diagnosis and surgical management [5,7,12]. Despite their benign nature, these cysts can cause pain, functional impairment, and aesthetic concerns, prompting patients to seek medical attention [13]. The case discussed in this paper exhibits atypical anatomical courses, which underscores the complexities associated with diagnosing and managing such cases. A review of the literature identified only one study describing a similar presentation of volar wrist ganglia originating from an atypical dorsal location. The previous study reported two cases of volar and radial ganglion cysts that originated from the dorsum of the scapholunate ligament [11]. This reinforces the importance of considering uncommon anatomical courses in the evaluation and surgical management of wrist ganglia to ensure complete excision and minimize recurrence.

Accurate imaging is crucial for the diagnosis and management of ganglion cysts. Ultrasound is commonly used due to its accessibility and ability to differentiate cystic structures from solid masses [14]. However, small ganglion cysts (≤10 mm) often appear hypoechoic without posterior acoustic enhancement, potentially leading to misinterpretation, and ultrasound may not always fully delineate the cyst’s extent, particularly when it extends into deeper anatomical planes [15]. In the presented case, the ultrasound confirmed the presence of a ganglion cyst adjacent to the flexor carpi radialis tendon, with no indication of a dorsal extension. 

Regarding managing ganglia, conservative management options, such as observation or aspiration, are often considered for asymptomatic or minimally symptomatic ganglia. However, aspiration has been associated with high recurrence rates, particularly for volar ganglia, due to their deeper location and proximity to vital structures, making surgical excision the preferred treatment modality in symptomatic cases [13,16].​ In this case, the decision to proceed with surgical excision was influenced by the patient's symptoms and the cyst's anatomical characteristics. Intraoperatively, the ganglion was found to extend dorsally, necessitating a dual-incision approach to ensure complete excision. This atypical presentation highlights the importance of thorough preoperative planning and the need for surgeons to be prepared for unexpected anatomical variations. Complete excision of the cyst, including its stalk, was crucial to minimize recurrence.

## Conclusions

This paper reports a case of a volar wrist ganglion cyst with an atypical anatomical course extending dorsally beyond its expected location. Despite the unusual presentation and the limitations of preoperative imaging, standard surgical excision resulted in the complete removal of the cyst and achieved a favorable clinical outcome.
